# A Rapid Biochemical and Radiological Response to the Concomitant Therapy with Temozolomide and Radiotherapy in an Aggressive ACTH Pituitary Adenoma

**DOI:** 10.1155/2017/2419590

**Published:** 2017-03-05

**Authors:** Ana Misir Krpan, Tina Dusek, Zoran Rakusic, Mirsala Solak, Ivana Kraljevic, Vesna Bisof, David Ozretic, Darko Kastelan

**Affiliations:** ^1^Department of Oncology, University Hospital Center Zagreb, Kispaticeva 12, 10000 Zagreb, Croatia; ^2^Zagreb University School of Medicine, Department of Endocrinology, University Hospital Center Zagreb, Kispaticeva 12, 10000 Zagreb, Croatia; ^3^Department of Endocrinology, University Hospital Center Zagreb, Kispaticeva 12, 10000 Zagreb, Croatia; ^4^Osijek University School of Medicine, Department of Oncology, University Hospital Center Zagreb, Kispaticeva 12, 10000 Zagreb, Croatia; ^5^Department of Radiology, University Hospital Center Zagreb, Kispaticeva 12, 10000 Zagreb, Croatia

## Abstract

*Background and Importance*. In the last eight years temozolomide (TMZ) has been used as the last-line treatment modality for aggressive pituitary tumors to be applied after the failure of surgery, medical therapy, and radiotherapy. The objective was to achieve a rapid control of tumor growth and hormone normalization with concurrent chemoradiotherapy in a patient with very aggressive ACTH pituitary adenoma.* Clinical Presentation*. We describe a patient with an aggressive ACTH-producing adenoma treated with concurrent temozolomide and radiotherapy. The patient suffered from an aggressive ACTH adenoma resistant to surgical and medical treatment. After two months of concurrent temozolomide and radiotherapy, cortisol normalization and significant tumor shrinkage were observed. After 22 months of follow-up, there is still no evidence of tumor recurrence.* Conclusion*. Concurrent treatment with temozolomide and irradiation appears to be highly effective in the achievement of the tumor volume control as well as in the control of ACTH secretion in aggressive ACTH adenoma.

## 1. Background and Importance

Pituitary adenomas are common, mostly benign tumors that are rarely subject to oncological treatment. In symptomatic or secretory pituitary adenomas the first-line treatment is the surgical removal of the tumor, which may be followed by medical therapy if no satisfactory results are achieved by surgery. Radiation therapy is often part of a multidisciplinary treatment of functional and nonfunctional tumors, usually as a third-line treatment after the failure of surgical and/or medical treatment. Radiotherapy is indicated for recurrent or progressive tumors after surgery, surgically inaccessible tumors (e.g., tumors extending to cavernous sinus), and biochemically uncontrolled tumors after maximal surgical and medical therapy, and it is also used as the treatment of choice for patients who are not candidates for surgery.

In the last eight years, temozolomide (TMZ) has increasingly been used as the last-line treatment for aggressive pituitary tumors resistant to conventional therapy [1–12]. TMZ is an oral alkylating agent approved for the treatment of glioblastoma. When used for aggressive pituitary tumors, TMZ is usually given in the conventional scheme including up to 12 cycles of therapy [13]. We report a case of an aggressive ACTH-producing pituitary adenoma in which a combination of radiotherapy and TMZ led to rapid biochemical, radiological, and clinical response.

## 2. Clinical Presentation

**Figure 1:**
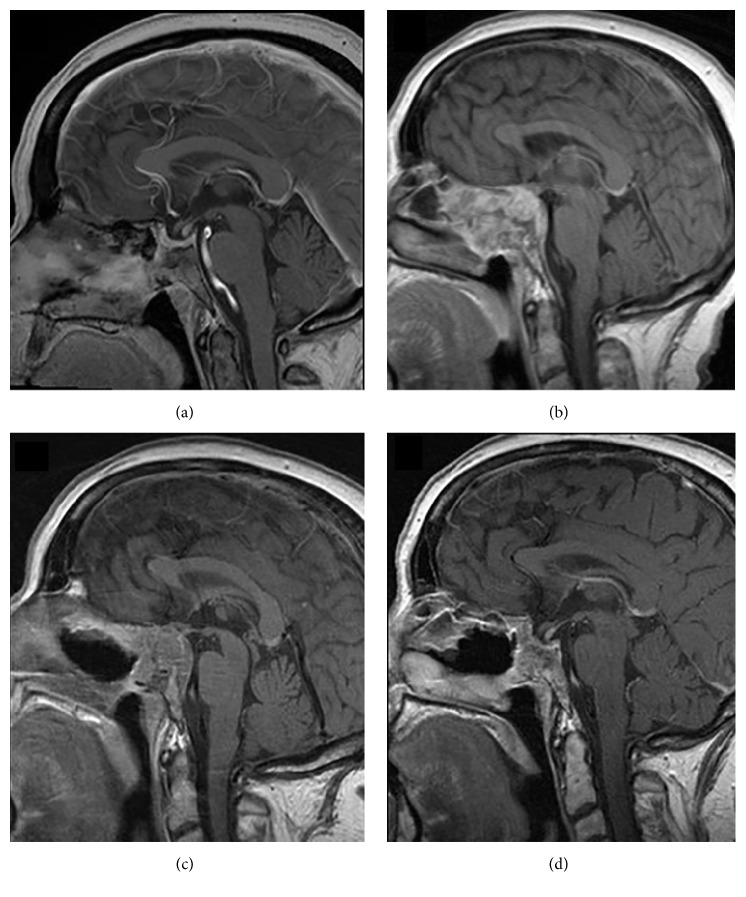
Pituitary MRI appearances (T1 postgadolinium weighted sagittal images). (a) After the first transsphenoidal operation for macrocorticotropinoma in 2010 with a small tumor remnant. (b) Three years after the first operation. Pituitary adenoma with destruction of the floor of the sella and invasion into sphenoid sinus and both cavernous sinuses. (c) Three months after the initiation of the concurrent therapy with TMZ and radiotherapy. (d) After the 6 cycles of TMZ. Stable pituitary remnant and biochemical control of the disease.

**Figure 2:**
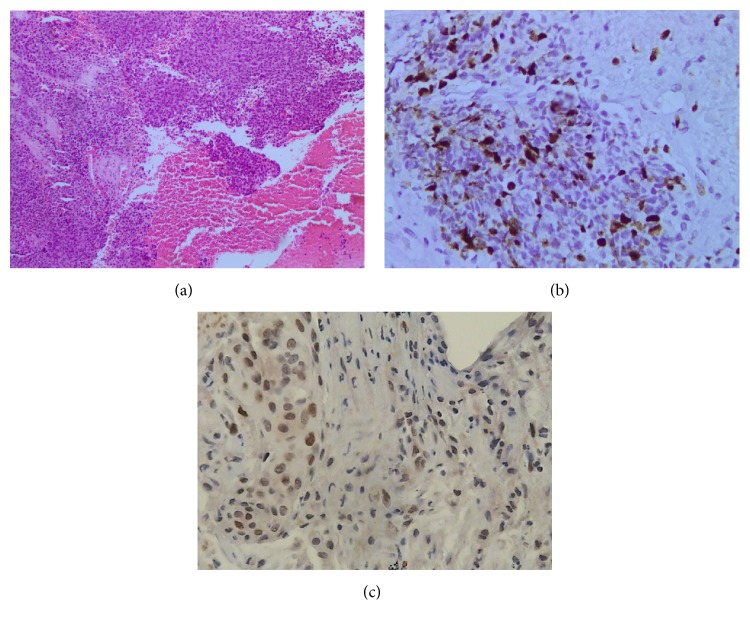
(a) HE staining of the pituitary macroadenoma tissue confirming atypical pituitary adenoma (magnification ×100). (b) Ki-67 positivity in tumor tissue of 10–20% (magnification ×400). (c) p53 positivity in less than 5% of cells (magnification ×400).

We report a 64-year-old female with Cushing's disease (CD). In April 2010, the patient first had a transsphenoidal surgery of a 15 mm large macrocorticotropinoma. The surgery led to biochemical remission with a presence of a small tumor remnant (Figure 1(a)). The tumor histology was consistent with atypical adenoma (Ki-67 20%, no mitoses, p53 not tested). Two years after the operation, tumor regrowth and biochemical relapse were observed. Urinary free cortisol and ACTH levels were 3,400 nmol/dU (NV < 369 nmol/dU) and 65.3 pmol/l (NV < 16 pmol/L), respectively. MRI confirmed tumor progression in sphenoid and ethmoid sinuses, both cavernous sinuses, with infiltration of the sellar wall, clivus, and chiasmal compression (Figure 1(b)). The patient suffered from headaches, visual field deficit, diplopia, ophthalmoplegia, and decreased visual acuity. The ketoconazole treatment was started. Transsphenoidal tumor reduction was performed, but severe hypercortisolism and ophthalmoplegia persisted. Postoperative MRI confirmed a large tumor remnant with infiltrative growth pattern destructing the bone (Figure 1(c)). The tumor tissue was positive for AE1/AE3, chromogranin, adrenocorticotropic hormone (ACTH), and growth hormone in some cells. Ki-67 was 10–20% and p53 positivity was present in less than 5% of cells (Figure 2). The repair enzyme O6-methylguanine-DNA methyltransferase (MGMT) was not determined. No distant metastases were found. The patient's general condition worsened with the right-side blepharoptosis, progressive visual impairment, and metabolic disturbances due to severe hypercortisolism.

Due to the rapid growth of the tumor remnant and the high value of the proliferation marker Ki-67, we decided not to wait for the effect of radiotherapy, but to immediately proceed with concurrent chemoradiotherapy (daily radiotherapy fractions of 2.0 Gy to a total dosage of TD 54 Gy concurrent with TMZ 75 mg/m^2^ per day). Radiotherapy was delivered by the linear accelerator and two opposed fields of 6/18 MV. Gross tumor volume (GTV) was 38.5 cm^3^ and planning target volume (PTV) 137.7 cm^3^. After two weeks, the patient's vision improved significantly with the recovery of the blepharoptosis and ophthalmoplegia, but she started to complain of weakness, dizziness, and fatigue. Low levels of morning cortisol (55 nmol/L (NR > 330 nmol/L)) were observed indicating biochemical remission of Cushing's disease and adrenocortical insufficiency. Ketoconazole was taken off and replacement therapy with hydrocortisone was started. The first follow-up MRI performed after 2 months of chemoradiotherapy showed the reduction of tumor volume of about 70%. We preceded with adjuvant TMZ in the dosage of 150 mg/m^2^ in the first cycle (from day 1 to 5) and 200 mg/m^2^ in the following five cycles (from day 1 to 5 every 28 days). The patient was in a significantly better clinical condition, on hydrocortisone replacement therapy and without major complaints or adverse events. The MRI after 3 and 6 months of therapy showed further tumor regression (Figure 1(d)). We decided to stop the treatment after 6 cycles and continued with a close follow- up. The last chemotherapy cycle was administered in January 2015. The patient tolerated the treatment very well, except for the fatigue reported from the beginning. Twenty-two months after the cessation of the TMZ treatment, the patient is still in remission of CD, with a stationary volume of the tumor remnant.

## 3. Discussion

ACTH-producing pituitary adenomas are generally benign tumors that are usually successfully treated with surgery. Medical therapy and radiotherapy are used in the case of surgical failure. In patients with CD, the overall tumor and hormone control rates in the reported studies are 97% and 74%, respectively, after a median follow-up of 8 years [14]. Fifty percent reduction in the urinary free cortisol level is usually observed 6 to 12 months after radiotherapy. It is estimated that the normalization of the serum cortisol level in patients with CD occurs about 24 months after radiotherapy [15]. The delay in the therapeutic response to radiotherapy is often unacceptable for some secretory, drug refractory tumors, as well as for aggressive tumors showing expansive growth.

Temozolomide is an orally available monofunctional DNA alkylating agent of the imidazotetrazine class. After spontaneous activation, it preferentially methylates DNA at N7 positions of guanine in guanine rich regions but also methylates N3 adenine and O6 guanine. There is a narrow pH window close to physiological pH at which the whole process of TMZ prodrug activation can occur. Brain tumors possess a more alkaline pH compared to surrounding healthy tissue, a situation which favors prodrug activation preferentially within tumor tissue. Methylation results in persistent DNA strand breaks, causing replication fork collapse. G2/M cell cycle arrest is triggered, occurring in the second cell cycle following treatment [16]. Both MGMT activity and mismatch repair (MMR) status of the tumor are important parameters that determine sensitivity to temozolomide [17].

In concomitant chemoradiotherapy, temozolomide reduces the number of cells in tumors undergoing radiation therapy by their independent cytotoxic action and by rendering tumor cells more susceptible to killing by ionizing radiation. Such drugs are potent enhancers of radiation response and thus might further improve the therapeutic outcome of chemoradiation therapy. The strategy of chemoradiation is to exploit the ability of chemotherapeutic agents to enhance tumor radioresponse. The enhancement denotes the existence of some type of interaction between drug and radiation at the molecular, cellular, or pathophysiologic level resulting in an antitumor effect greater than would be expected on the basis of additive actions. Temozolomide makes damaged DNA more susceptible to radiation damage resulting in enhanced cell killing [18]. In recent years the use of TMZ has been reported in aggressive pituitary tumors [1–12]. Raverot et al. reported 18 patients with ACTH tumors treated with TMZ. After 9.1 ± 4.7 cycles of therapy, biochemical response, defined as a 50% decrease in ACTH secretion, was observed in 67% of patients. In the same study, a reduction in tumor volume, defined as a 20% decrease in maximal tumor size, was observed in 56% of patients. According to the results of different studies, tumor shrinkage or hormonal response to temozolomide treatment is usually observed within weeks after treatment initiation in responding patients [19]. Treatment regimens with TMZ in pituitary tumors are variable, but the one most frequently used is the conventional regimen with 150–200 mg/m^2^/day from days 1 to 5 every 28 days [20].

In the majority of the reported cases, TMZ has been used after the exhaustion of all the three treatment modalities (surgery, medical therapy, and radiotherapy). However, due to the rapid tumor growth resulting in the mass effect and uncontrolled hypercortisolism, we decided to apply a more aggressive therapeutic strategy using TMZ together with radiotherapy. Such a treatment regimen is usually applied in the treatment of high-grade glioma [21], which was the rationale for the choice of the treatment in the case of the aggressive pituitary adenoma in question. Besides the expected synergistic effects of the two different treatment modalities (TMZ and radiotherapy), a possible disadvantage of concurrent chemoradiotherapy is that it bears the risk of increased toxicity and side effects.

In our patient the effect of the combination of TMZ and radiotherapy was unexpectedly fast and led to rapid tumor shrinkage as well as to rapid control of hypercortisolism. The excellent therapeutic response could probably be attributed to the tumor histology consistent with atypical adenoma characterized by high proliferative indices and rapid cell division. Until now, there has only been one published case report on the use of the concurrent radiotherapy and TMZ in an aggressive nonfunctional adenoma, also showing good therapeutic results [22]. Therefore, we might speculate about the possible potentiation of the radiation effect by TMZ in aggressive pituitary adenoma as reported in relation to glioblastomas [23]. We might also hypothesize that concurrent chemoradiotherapy has a promising role in the treatment of selected cases of rapidly growing, aggressive pituitary corticotropinoma with high proliferation indices. Since temozolomide has low toxicity, good tolerability, and two decades of proven efficacy in other brain tumors, the concurrent chemoradiotherapy should be considered earlier in the course of the disease.

## 4. Conclusion

The concurrent use of TMZ and radiotherapy appears to be a helpful alternative for the treatment of rapidly growing, aggressive pituitary ACTH-producing adenomas resistant to conventional treatment.
